# Characterization of the Oral Microbiome of Medicated Type-2 Diabetes Patients

**DOI:** 10.3389/fmicb.2021.610370

**Published:** 2021-02-05

**Authors:** Ana Almeida-Santos, Daniela Martins-Mendes, Magdalena Gayà-Vidal, Lucía Pérez-Pardal, Albano Beja-Pereira

**Affiliations:** ^1^Centro de Investigação em Biodiversidade e Recursos Genéticos (CIBIO-UP), InBIO, Universidade do Porto, Porto, Portugal; ^2^Department of Geosciences, Environment and Spatial Planning (DGAOT), Faculty of Sciences, University of Porto, Porto, Portugal; ^3^Internal Medicine Department, Centro Hospitalar de Vila Nova de Gaia/Espinho EPE, Vila Nova de Gaia, Portugal; ^4^Diabetic Foot Clinic, Endocrinology, Diabetes and Metabolism Department, Centro Hospitalar de Vila Nova de Gaia/Espinho EPE, Vila Nova de Gaia, Portugal; ^5^Department of Biomedicine, Faculty of Medicine, University of Porto, Porto, Portugal; ^6^i3S–Instituto de Investigação e Inovação em Saúde, Universidade do Porto, Porto, Portugal; ^7^Faculty of Sciences, Sustainable Agrifood Production Research Centre (GreenUPorto), University of Porto, Porto, Portugal

**Keywords:** type 2 diabetes mellitus, 16S rRNA gene sequencing, microbiota, next-generation sequencing, Portugal, oral hygiene

## Abstract

Type 2 diabetes mellitus (T2DM) is a chronic metabolic disease that is becoming a significant global health care problem. Several studies have shown that people with diabetes are more susceptible to oral problems, such as periodontitis and, although the causes are still inconclusive, oral microbiota is considered to play a major role in oral health. This study aimed to characterize the oral microbiome of a sample representing T2DM patients from Portugal and exploit potential associations between some microorganisms and variables like teeth brushing, smoking habits, average blood sugar levels, medication and nutrient intake. By sequencing the hypervariable regions V3-V4 of the 16S rRNA gene in 50 individuals belonging to a group of diabetes patients and a control group, we found a total of 232 taxa, from which only 65% were shared between both groups. No differences were found in terms of alpha and beta diversity between categories. We did not find significant differences in the oral microbiome profiles of control and diabetes patients. Only the class *Synergistia* and the genus *TG5*, which are related to periodontitis, were statistically more frequent in the control group. The similar microbiome profiles of medicated diabetics and the control group indicates that the relationship between the T2DM and the oral microbiome might be more related to either the lifestyle/diet rather than diabetes *per se*. Moreover, this study provides, for the first time, insights into the oral microbiome of a population with a high prevalence of diabetes.

## Introduction

Type 2 diabetes mellitus (T2DM) is a metabolic disease characterized by chronic hyperglycemia caused by defects in insulin secretion, which can contribute to the development of resistance to its action (Kuo et al., 2008). T2DM is becoming more common, also affecting children, and therefore, represents a significant global health care problem (Issa, 2017). According to the International Diabetes Federation, the prevalence of T2DM in Portugal is 9.8% whereas the prevalence in Europe is 6.3% and 8.81% worldwide (International Diabetes Federation, 2019).

A growing number of studies have been reporting a close association between diabetes and susceptibility for some oral illnesses, such as periodontitis (Mealey et al., 2006), derived from the deregulation of the oral microbiota equilibrium that increments the establishment of pathogenic organisms, causing the deregulation of the oral microbiota equilibrium, and vice-versa.

The oral microbiota is one of the most diverse and dynamic ecosystems of the human body, in which more than 700 species of bacteria have been identified (Long et al., 2017). Bacterial phyla *Firmicutes, Actinobacteria, Fusobacteria, Proteobacteria*, and *Bacteroidetes* dominate the oral microbiota, accounting for 80–95% of the total species (Yang et al., 2012). These microorganisms normally harmoniously co-exist with their host due to coevolution, however, behavioral factors such as poor oral hygiene and diet, debilitated immune systems, genetics, medication and, certain diseases can lead to a dysbiotic oral ecosystem (Nath and Raveendran, 2013). This imbalance is normally associated with an overgrowth of pathogenic microorganisms, which can lead to more susceptibility to oral illness (Woelber et al., 2016). Factors as diet, lifestyle, age, medication, denture wear, saliva flow, several diseases, and a poor immune system tend to affect the microbiome composition (Ticinesi et al., 2018). In addition, the oral cavity is a heterogeneous environment due to the variety of distinct habitats (i.e., teeth, gingival sulcus, tongue, cheeks, hard, and soft palates), each of them with a particular microbiota. Saliva is a non-invasive and easy collectable biological material, which microbiome is partially shared with that of all different sites of the buccal cavity due to contact, and therefore is a good representative to investigate the oral microbiome (Takeshita et al., 2016).

The oral microbiota plays an important role in the relationship between periodontitis and diabetes (Kononen et al., 2019) since it influences glycemic control (Taylor et al., 1996). Certain bacteria such as *Porphyromonas gingivalis*, one of the main strains of periodontal disease, triggers periodontal tissue destruction and increases insulin resistance (Kuo et al., 2008). There are more taxa related to T2DM, Long et al. (2017) compared the oral microbiome profiles from African Americans subjects with T2DM with non-diabetic obese individuals and non-diabetics with normal-weight. They found that a higher abundance of taxa in the phylum *Actinobacteria* (*Actinomycetaceae, Bifidobacteriaceae, Coriobacteriaceae, Corynebacteriaceae, and Micrococcaceae* families) were associated with lower diabetes risk, as they were less abundant among diabetic subjects compared to normal-weight controls. Differences in taxa proportions were also found comparing diabetics and non-diabetics in subjects with caries in Indians (Latti et al., 2018) and periodontitis in Brazilians (Casarin et al., 2013). Another study found a decrease in the biological and phylogenetic oral microbiome diversity in diabetics in comparison to non-diabetics from South Arabia, evidence that was related to an increase in the pathogenic content in the diabetic’s oral microbiome (Saeb et al., 2019). In contrast, others (Kampoo et al., 2014) did not find diversity differences between T2DM and control samples from Thailand.

Presently, the only two studies on the relationship between T2DM and the oral microbiome made on European populations used a limited sample size (*n* < 20) and were focused on the subgingival microbiome (Farina et al., 2019) or obese T2DM subjects (Tam et al., 2018). But the oral microbiome is an important factor for the maintenance of human health, and thus disentangling the relationship between diabetes and oral microbiome is of paramount interest. Here, we characterized the oral microbiome from a sampling of medicated patients from Portugal and compared them with a control group, by using next-generation amplicon sequencing of the hypervariable V3-V4 regions of the 16s rRNA from saliva samples of 50 individuals.

## Materials and Methods

### Sampling and Questionnaire Administration

Twenty-five patients with T2DM (average age 63) and twenty-five healthy individuals (average age 60) participated in this study (Supplementary Table S1). The study was approved by Centro Hospitalar de Vila Nova de Gaia/Espinho’s Ethics Committee and conducted according to the Declaration of Helsinki. Written informed consent was acquired from all participants before sampling.

Volunteers were ineligible if they presented less than one-third of the dentition, were under 18 years old, or had taken antibiotics less than 2 months before. All participants were instructed not to brush their teeth after their last meal before the saliva sample collection. In addition, each volunteer was submitted to a questionnaire, through face-to-face interviews, regarding their lifestyle, including information on smoking and oral hygiene habits (Supplementary Table S1), food restrictions, health status, diabetes duration (in years), types of medications they were taking (Supplementary Table S2) and the period time in the case of antibiotics. Also, average blood sugar levels were collected through the hemoglobin A1c test (HbA1c) (Supplementary Table S1). Teeth brushing habits were divided into 4 categories: brushing: (i) > once/day, (ii) once/day, (iii) 1–3 times/week, and iv) never brushing. Mouthwash use was divided into 3 categories: 1–3 times per week, 1 per day, and no use. Smoking habits were classified into heavy smokers, moderate smokers, and non-smokers. BMI was divided into 6 categories: underweight, normal weight, preobese, obese (I, II, III). The variable age was divided into 3 categories according to percentiles (0–33% percentile, 33–67% percentiles, and 67–100% percentiles) and sex into male or female. Diabetics HbA1c levels were classified in 2 categories: (i) controlled (HbA1c < 7) and (ii) poorly controlled (HbA1c > 7). All diabetic patients were prescribed insulin and/or oral antidiabetic medication. We examined a total of 11 drugs categories on diabetics patients: (i) Metformin, (ii) Proton pump inhibitors (PPIs), (iii) Dipeptidyl Peptidase 4 (DPP4) inhibitor, (iv) Sodium-glucose co-transporter 2 (SGLT2) inhibitor, (v) levothyroxine, (vi) Sulfonylurea, (vii) Statin, (viii) Antidepressants, (ix) statin + metformin, (x) DPP4 inhibitor + metformin, and (xi) insulin (Supplementary Table S3).

A validated semiquantitative food frequency questionnaire (Lopes, 2000; Lopes et al., 2007) was used to estimate nutrient intake. Nutrient content was calculated using the Food Processor Plus (ESHA Research, Salem, Oregon) program. The consumption of some nutrients such as protein, carbohydrates, monounsaturated, polyunsaturated, saturated and total fat, sugar as calorie intake can be found in Supplementary Table S4.

### DNA Extraction

DNA was extracted from saliva following Quinque et al. (2006) and quantified using a NanoDrop 2000 spectrophotometer (Thermo Fisher Scientific Inc., MA, United States).

### 16S rRNA Amplification, Library Preparation, and Sequencing

To amplify the V3-V4 hypervariable regions, the 341F/805R universal primers were used (Iriboz et al., 2018). A two-step Polymerase Chain Reaction (PCR) was used to first amplify the target region and then to attach a barcode to each sample before pooling them for illumina sequencing. The amplicon PCR contained 5 μl of DNA template at a concentration of 10 ng/μl, 5 μl of Taq PCR Master Mix kit (Qiagen), 0.4 μl of each primer (100 pmol/μl) and 3.2 μl of distilled and deionized water, in a final reaction volume of 14 μl per sample. The PCR cycling conditions were 95°C for 15 min, followed by 40 cycles of denaturation at 95°C for 30 s, annealing at 55°C for 1 min, and elongation at 72°C for 30 s. The final elongation was run at 60°C for 5 min, followed by a hold at 15°C.

The amplicons’ size was checked in 2% agarose gel and purified using the AMPure XP kit according to the manufacturer’s instructions. A second PCR was performed using two indices (i5 and i7) with 7 bp each based on McInnes et al. (2016). The reaction contained 5 μl of 2× Kapa HiFi Hot Start, 0.5 μl of each index, 2 μl of ultrapure water and 2 μl of first PCR product DNA, in a final volume of 10 μl per sample. PCR cycling conditions were run at 95°C for 3 min, succeeded by 10 cycles of denaturation at 95°C for 30 s, annealing at 55°C for 30 s, and elongation at 72°C for 30 s. The indexed amplicons were purified using the AMPure XP kit according to the manufacturer’s instructions followed by library quantification using a Qubit^TM^ dsDNA BR Assay Kit (Thermo Fisher Scientific). All samples along with two negative controls were normalized to 9 nM and pooled with 5 μl of each sample. The two negative controls correspond to an extraction blank and a library blank.

A TapeStation 2,200 (Agilent Technologies) was used for the precise sizing and library quantification of the pool, followed by a library validation through a quantitative PCR. Finally, the pool was sequenced in an Illumina MiSeq sequencing platform, using the MiSeq v2 500-cycle sequencing kit (Illumina Inc.), with a 2 × 250 bp paired-end configuration at Novogene facilities. Raw metagenomic data are available from the SRA database with accession number PRJNA679485.

### Sequence Processing and Alignment

Reads were analyzed using the Quantitative Insights into Microbial Ecology pipeline (QIIME) version 2-2019.7 (Bolyen et al., 2019). The paired-end sequences were processed through DADA2 (Callahan et al., 2016), a quality control package in QIIME2, following the workflow: filtering, denoising, dereplication, chimera identification, and merging. The resulting output was a feature table with the quantity of each amplicon sequence variant (ASV) in each sample. Three diabetic samples were discarded due to the low number of reads.

For the taxonomic assignment, we used the Naïve Bayes classifier trained on the Greengenes (version 13.8) (DeSantis et al., 2006). The ASVs annotated as mitochondria and chloroplast were removed. One control sample was discarded due to an excessive number of reads, being 90% of them assigned to a taxon unreported in the oral microbiome, probably reflecting a sequencing artifact.

### Statistical Analyses

The taxonomic abundance and ASV relative frequencies were calculated for each sample with QIIME2, for the phylum, class, genus, and species level. The differential taxa abundance between the two groups was evaluated through the Analysis of Composition of Microbiomes (ANCOM) and the Mann-Whitney U-test in SPSS v.25, which was also used to compare the relative frequencies of bacteria associated with the periodontitis between the control and diabetes groups. To perceive if potential differences between the microbiome of both groups could be due to the diet, nutrients consumption and energy intake were compared between groups with the Mann-Whitney U-test or a Student’s *t*-test (in normal distribution data), with a 5% level of significance, followed by a Bonferroni correction for multiple tests.

Microbiome diversity was evaluated within individuals (alpha-diversity) with the ASVs abundance and the Shannon diversity index (Shannon, 1948), and between samples (beta-diversity) through the Bray-Curtis dissimilarity (Bray and Curtis, 1957), which considers abundance data, and through the Jaccard distance (Jaccard, 1908), which considers absence/presence data. For the alpha diversity, we performed rarefaction curves with QIIME2 and all samples were rarefied to a depth of 1973 reads. Differences in alpha diversity between the control and diabetes groups, as well as between categories of teeth brushing, mouthwash use, smoking habits, BMI, sex, age, HbA1c levels, and types of medication were evaluated by the non-parametric Kruskal-Wallis H-test in QIIME2. The variables teeth brushing, smoking habits and BMI were divided into new categories in order to increase the statistical power: teeth brushing (<1 per day, 1 per day, >1 per day), smoking habits (smokers and non-smokers) and BMI (normal weight, preobese, obese).

Spearman’s correlation test was used to investigate the correlation between the diabetics’ HbA1c levels and the obtained taxa using the SPSS V.25.

Lastly, to investigate the microbiome composition profiles of diabetics and controls, we explored the grouping patterns with: (i) a principal coordinate analysis (PCoA) based on the Bray-Curtis matrix, performed through EMPeror (Vazquez-Baeza et al., 2013), and (ii) a hierarchical clustering and a heatmap performed with the pheatmap package (Kolde, 2019) in R (v3.5.1) (R Core Team, 2017) based on taxa abundance frequencies using only the taxa present in more than 15% of the samples. Besides, we tested whether the microbiome composition was statistically different between both groups using the Permutational Multivariate Analysis of Variance (PERMAVONA) analysis (Anderson, 2001) with 999 permutations based on the Bray-Curtis matrix.

## Results

### Sequencing Data and Taxonomic Assignment

A total of 12,754,645 raw reads were obtained with a mean of 255,092.9 reads per sample (range: 147–1,085,170). No reads were generated from the library blank or extraction blank. After quality filtering and mitochondria and chloroplast removal, 752,526 reads remained for further analyses, with an average of 15,053 reads per sample.

Considering the 46 samples included in the analysis, the high-quality reads were assigned to 10,746 ASVs with a total absolute frequency of 605,211. The median ASV frequency per sample was 12,437, with a mean of 13,144.59 (range: 1,973–35,935). The ASVs were assigned to 232 taxa (Supplementary Table S5). The taxa identification was possible for 71% of the ASVs at the genus level, and 21% at the species level. Control and diabetes groups shared 153 out of the 232 taxa. The control group exhibited 50 taxa not present in the diabetes group and the diabetes group presented 31 taxa absent in the control.

### Microbiome Characterization

We detected a total of 13 phyla, 21 classes, 37 orders, 60 families, 86 genera, and 51 species. At the phylum level, the oral cavity of all the 46 samples was dominated by Firmicutes (45%), Bacteroidetes (22%), Proteobacteria (16%), Actinobacteria (9%), and Fusobacteria (6%), constituting 98% of the total oral microbiome.

At the class level, all individuals exhibited an average of 14 taxa. The 10 most frequent classes accounted for ∼97% of the total abundance in both groups (Supplementary Figure S1). *Bacilli* and *Bacteroidia* are the dominant classes in both groups, accounting for 50%. *Gammaproteobacteria* had a higher abundance, not significant (*p* = 0.241), in the control (9.9%) than in the diabetes group (5.4%) (Supplementary Figure S1). Five classes were significantly different between the two groups, *Betaproteobacteria* was higher in the diabetics group (*p* = 0.033), and *Deltaproteobacteria* (*p* = 0.013), *Spirochaetes* (*p* = 0.035), *Mollicutes* (*p* = 0.043), and *Synergistia* (*p* < 0.001) were higher in the control group, nevertheless, after Bonferroni correction, only *Synergistia* class remained significantly (Supplementary Table S6).

**FIGURE 1:**
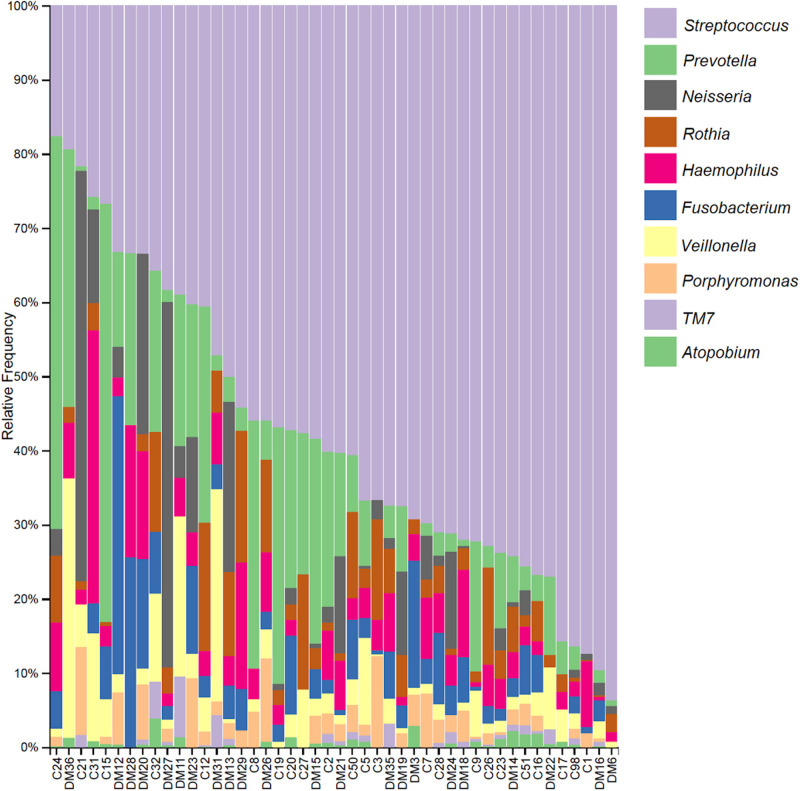
Relative frequency of the 10 most abundant taxa up to the genus level per subject. Sample names starting with C are from the control group and those starting with DM are from the diabetics group.

When focusing on the taxa abundance at the genus level, all individuals displayed an average of 32 genera. The frequency distribution of the 10 most abundant taxa, up to genera is shown in Figure 1. *Streptococcus* (29%) is the most abundant genus, present in all subjects, followed by *Prevotella* (14%) and *Neisseria* (5%).

**FIGURE 2:**
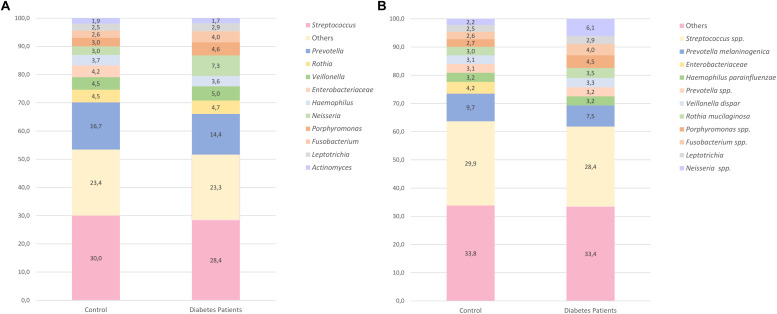
Relative abundance of the 10 most-abundant taxa found at the genus level **(A)** and at the species level **(B)** in both control and diabetes groups. The remaining taxa are included in the category “Others”.

The 10 most frequent genera accounted for approximately 77% in both groups (Figure 2A). Some of the taxa were identified only up to the family level due to lack of resolution. Between the control and diabetes groups, 14 taxa were statistically different (Supplementary Table S6). Nonetheless, after the Bonferroni correction, only the *TG5* genus remained significant (*p* < 0.001). *TG5* genus belongs to the *Synergistia* class, the only class that was statistically significant.

At the species level, an average of 16 different taxa per individual was observed (most taxa were not identified up to species level). The 10 most-frequent taxa (only four were assigned to species level), accounted for 67% in both groups (Figure 2B). *Streptococcus* spp. and *Prevotella melaninogenica* were the most frequent species in both. For the two studied groups, 20 taxa were significantly different (Supplementary Table S6), but after Bonferroni correction, only *TG5* spp. remained statistically significant, being more abundant in the control group. The ANCOM analysis of ASV differential abundance also revealed a significantly higher abundance of the *TG5* genus and *TG5* spp. in the control group (*p* < 0.001).

### Diversity Measures

#### Alpha Diversity

**FIGURE 3:**
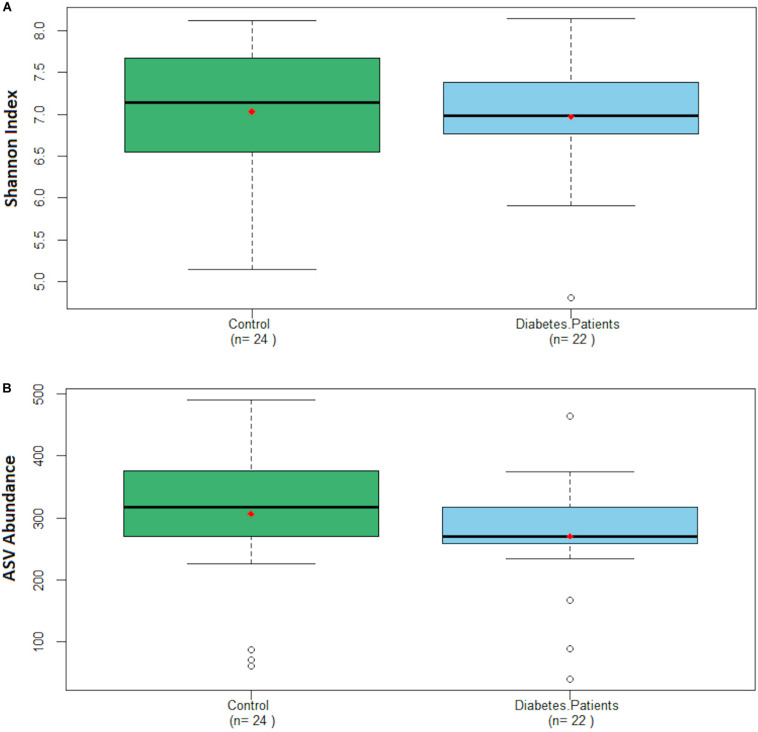
Boxplot charts depicting the distribution of the Shannon index **(A)** and ASV abundance **(B)** regarding both groups. Red dot represents the mean of each group.

The Shannon index was 7.03 ± 0.75 for the control group and 6.97 ± 0.66 for the diabetes group, whereas the ASVs abundance was 306 ± 111 and 271 ± 86 for the control and the diabetes group, respectively (Figure 3). We did not find significant differences in the distribution of the Shannon index nor the ASVs abundance of both groups.

#### Beta Diversity

**FIGURE 4:**
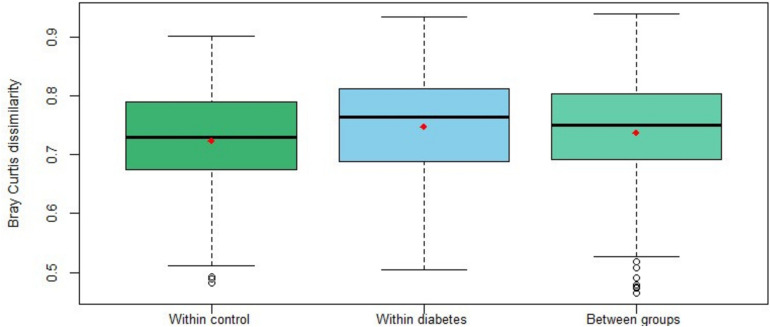
Distribution of pairwise Bray-Curtis dissimilarity values between individuals within diabetics and controls and between them. The red color dots represent the mean of each distribution.

Bray-Curtis dissimilarity values were calculated to measure the differences between individuals, in terms of taxonomic structure. Figure 4 shows the distribution of the pairwise Bray-Curtis dissimilarity values between individuals within each group and between them, and similar distributions are observed. This was confirmed with the PERMANOVA analysis (pseudo-*F* = 1.092; *p* = 0.231) indicating that there is no differentiation in the microbiome composition of the diabetes group compared to the control one. Similar results were obtained with the Jaccard distance values (pseudo-*F* = 1.053; *p* = 0.178).

**FIGURE 5:**
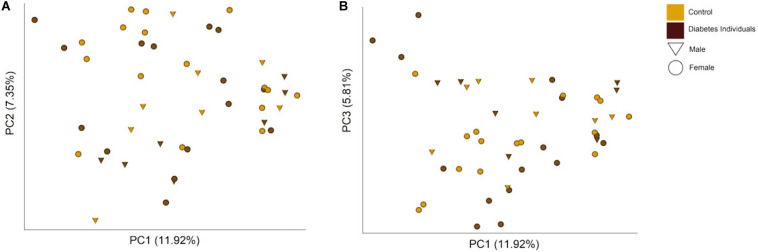
PCoA plots showing the **(A)** first and second, and **(B)** first and third principal components and the percentage of the total variance that they explain based on the Bray Curtis dissimilarity matrix. Each point represents one individual, with color and symbol indicating the group of study and sex.

The differences between the microbiome profiles of the individuals were visualized through a Principal-Coordinates Analysis (PCoA), from the Bray Curtis dissimilarity matrix, that did not reveal a clear clustering pattern (Figure 5). The hierarchical clustering analysis formed two major clusters (Supplementary Figure S2). The clusters seem to be determined mainly by the *Streptococcus* abundance and secondly by *Prevotella melaninogenica* and *Veillonella dispar* and to a lesser extent possibly by *Neisseria* and *Rothia mucilaginosa* abundances. Both clusters contain individuals from both groups.

### Bacteria Associated With Periodontal Disease

Due to the relationship between Diabetes and periodontal disease, we compared the relative abundance of species that have been related to periodontal disease (*Prevotella intermedia, Campylobacter rectus, Porphyromonas endodontalis, Treponema socranskii*, and *TG5* spp.) (Rams et al., 1993; Takeuchi et al., 2001; Lombardo Bedran et al., 2012; Vengerfeldt et al., 2014), being vestigial in both groups. *TG5* spp. was the only species statistically different between groups after the Bonferroni correction, presenting more abundance in the control group (Supplementary Table S4).

### Oral Hygiene, Smoking Habits, and Demographic Data

We evaluated how these habits affected the oral microbiome of the individuals studied. Regarding the alpha diversity (Supplementary Figure S3), our results showed that individuals who brush their teeth once per day have a higher Shannon index and ASVs abundance followed by those who brush more than once per day.

Concerning smoking habits, smokers showed a higher Shannon index, as well as ASVs, when compared to non-smokers.

As to the variable age, those from the percentile 33–67% presented the highest Shannon index value, however, the percentile 67–100% was the one with higher ASVs abundance.

Females have a higher Shannon index as ASVs abundance than males. Additionally, considering the BMI variable, the obese category has a higher Shannon index as ASVs abundance, followed by the preobese category.

However, none of the comparisons between the above-mentioned categories were statistically significant.

The PCoA plot colored by the teeth brushing and smoking habits’ categories (Supplementary Figures S4, S5) did not reveal a clear clustering pattern. Likewise, none of the individual aggregation in the hierarchical clustering was attributable to a particular habit.

#### HbA1c Levels and Medication on Diabetics Patients

HbA1c levels were positively correlated with *Streptococcus* (*p* = 0.042; *P* = 0.470), *Granulicatell*a (*p* = 0.014; *P* = 0.554) and *Lautropia* (*p* = 0.010; *P* = 0.575) genera and negatively correlated with *Oribacterium* (*p* = 0.007; *P* = −0.600), and *Catonella* (*p* = 0.038; *P* = −0.479) genera. After the Bonferroni correction test, only *Lautropia* and *Oribacterium* genera remained statistically significant.

We did not find significant differences in the distribution of the Shannon index for the HbA1c categories (Supplementary Figure S6) nor for the 11 drug categories (data not shown).

The PCoA plot dyed by the glycemic level categories (Supplementary Figure S7) and by the 11 drug categories (Supplementary Table S3) in diabetics patients did not disclose a clustering pattern in any of the cases.

### Diet

We measured nutrient intake to be able to identify differences in the oral microbiome of diabetes and controls that could be related to diet. However, no significant differences were found regarding nutrients consumption and energy intake between both groups after Bonferroni correction (Table 1).

**TABLE 1:** Description of the participants per group of study and respective *p*-values of Mann-Whitney test/Student’s *t*-test between the groups when applicable.

		Control	Diabetes patients	
		Mean ± SD	Mean ± SD	*p*-value
Protein		94.5 g/day ± 32.2	111.1 g/day ± 35.4	0.043*
Carbohydrates		218.5 g/day ± 70.9	236.2 g/day ± 75.0	0.839
Sugar^t^		92.5 g/day ± 31.5	84.0 g/day ± 32.6	0.352
Total fat		73.4 g/day ± 27.2	85.0 g/day ± 35.8	0.432
Monounsaturated fat		34.4 g/day ± 13.2	40.4 g/day ± 19.0	0.421
Polyunsaturated fat		12.4 g/day ± 4.7	15.3 g/day ± 7.5	0.607
Saturated fat		20.4 g/day ± 8.7	21.9 g/day ± 8.0	0.421
Calories		1946.7 Kcal/day ± 584.1	2199.8 Kcal/day ± 726.4	0.594
BMI		27.1 kg/m^2^ ± 4.5	28.4 kg/m^2^ ± 5.1	
Age		60.0 years ± 8.8	62.7 years ± 7.0	
HbA1c		−	7.2% ± 1.0	
Diabetes duration (years)		−	14.5 years ± 10.6	

		**N**	**N**	

Smoking Habits	Heavy smokers	3	1	
	Moderate Smokers	2	2	
	Non-smokers	20	22	
Teeth brushing	1–3 per week	3	4	
	1 per day	5	11	
	>1 per day	17	6	
	No use	0	4	
Mouthwash use	1–3 per week	11	4	
	1 per day	1	5	
	No use	13	16	
Anti-diabetic drugs	Insulin	−	8	
	DPP4 inhibitor	−	11	
	SGLT2 inhibitor	−	6	
	Metmorfin	−	17	
	Sulfonylurea	−	3	

**This information can also be found per individual in Supplementary Table S1.****^∗^****Statistically significant. However, after Bonferroni correction, the adjusted p-value was 0.387.****^*t*^****Variables to which Student’s t-test was applied. The remaining variables were analyzed using the Mann-Whitney test.**

## Discussion

This study aimed to characterize the oral microbiota in diabetic individuals from Portugal using the 16S rRNA sequencing method to shed light on the relationship between the oral microbiome and T2DM. For the first time, the oral microbiome composition of a Portuguese population was disclosed. The predominant phyla are in line with previous studies from other populations (Yang et al., 2012; Verma et al., 2018). Likewise, the taxa frequency distribution found in our samples follows the pattern usually observed in the oral microbiome, in which few taxa recruit most sequences (e.g., Gomez et al., 2017; Willis et al., 2018). For example, Keijser et al. (2008) study on healthy adults reported that *Prevotella, Streptococcus, and Veillonella* genera were responsible for about 50% of the total salivary microbiome, which is similar to our findings. Although at very low frequencies (0.25–0.87%), we found the *Gluconacetobacter* genus in nine individuals. Interestingly, this genus is not present in the Human Oral Microbiome Database (Chen et al., 2010), nor described, as far as we know, in other oral microbiome studies. Species of this genus have been found in plants, grapes and wine spoilage (Bertalan et al., 2009; Campaniello and Sinigaglia, 2017), and neoplastic tissue in breast cancer (Hieken et al., 2016). A possible explanation for this finding might be the presence of food remains in the saliva. Another interesting finding was the low average number of taxa at the species level per individual (16) when compared to similar studies (∼200–600 species) (Saeb et al., 2019; Schulz et al., 2019). This is probably because the 16s RNA fragment used has a relatively low capacity to discriminate within the lowest taxonomic levels (genus and species). For this reason and the fact that we did not confirm the taxonomic identification at species level by quantitative PCR, we recommend caution while considering the species identified in this study.

As the oral microbiome diversity may decrease with frequent oral hygiene habits (Pyysalo et al., 2019), we further investigated the influence of oral hygiene and smoking habits on microbiome diversity. According to some studies, those who brushed their teeth twice per day presented a lower diversity than those who brushed it more rarely (Pyysalo et al., 2019). However, our results showed no differences between the three categories. Concerning mouthwashes, it would be expected a decrease in biodiversity with the increase of its use, since most of the mouthwashes present antimicrobial properties (Okuda et al., 1998; Tribble et al., 2019), nevertheless, our results did not show differences between those who use it and those who not. As for smoking habits, although previous studies reported that smokers tend to have a more diverse microbiota, including pathogenic taxa, than non-smokers (Takeshita et al., 2016), our results did not show differences between smokers and non-smokers.

With respect to diabetics’ average blood sugar levels, we did not find differences in terms of microbiome diversity between the controlled and poorly controlled groups. The same results were found by Tam et al. (2018) when studied the glycemic level of obese type 2 diabetics. Nonetheless, we found associations between the HbA1c and *Lautropia* and *Oribacterium* genera being the first genus commonly found in the oral cavity and the second is considered a biomarker of oral and liver cancer (Chattopadhyay et al., 2019; Lu et al., 2019).

Another factor described in the literature that affects the microbiome composition and diversity is medication. Here, we analyzed the possible effects of type 2 diabetes medication as well as other drugs non-related to diabetes, which according to the literature, can affect the gut microbiome. Drugs used to treat type 2 diabetes such as Metformin, were reported to modify the gut microbiome, whereas information about DPP4 and SGLT2 inhibitors is lacking (Hung and Hung, 2020).

Additionally, drugs such as PPis, used to decrease stomach acid production have been reported to induce gut microbiome dysbiosis (Bruno et al., 2019) as to an increase in common oral bacteria in the gut (Imhann et al., 2016). In our study, none of the drugs tested, showed differences related to microbiome diversity. As far as we know, there are no studies regarding the influence of drugs in oral microbiota.

Regarding the comparison of the oral microbiome of diabetics and the control group, overall, the control group showed a higher, although not significant, amount of taxa (202) than the diabetics (183), a trend that is in line with previous studies using saliva samples (Sabharwal et al., 2019; Saeb et al., 2019). Other studies on subgingival plaque reported the opposite (Casarin et al., 2013) in diabetes individuals not controlled by medication, a factor that could explain these differences.

When we focus on the abundance differences of particular taxa, our results showed that *Actinobacteria* was slightly more frequent in the control group, although not statistically significant. This tendency is consistent with Long et al. (2017), where *Actinobacteria* was associated with a decreased risk of diabetes. We did not find differences between both groups for the most abundant classes, *Bacilli*, and *Bacterioidia*, in contrast to Saeb et al. (2019). These authors found that both classes were more abundant in the normal glycemic group compared to T2DM individuals. On the other hand, and in line with Saeb et al. (2019), in our study, *Gammaproteobacteria* abundance was higher but not significant in the healthy individuals, whereas *Betaproteobacteria* was significantly higher in the diabetes group (*p* = 0.03). An interesting result was that the *TG5* genus (*Synergistia* class), the only one that showed significant differences between groups, has been related to periodontitis (Vengerfeldt et al., 2014), failed implants (Dingsdag et al., 2016), and smoking habits (Valles et al., 2018). In our findings, this taxon was found in 72% of the healthy samples against only 9% of diabetes individuals. However, the frequencies of this and other genera related to periodontitis, and thus, to diabetes (Preshaw et al., 2012), are vestigial in our groups, being the *TG5* just slightly higher in controls. As at the moment of sampling we did not conduct a detailed medical evaluation of the individuals’ periodontal/oral health, and only inquired the individual on the last year history of its periodontal/oral condition, we could not conduct association test between oral health and the presence/absence of a specific microorganism.

Concerning the association between T2DM and the oral microbiome, we did not find clear differences between the microbiome composition of both groups, even though it was possible to identify some taxonomic dissimilarity. The work of Sabharwal et al. (2019) using saliva samples from controlled diabetics from the United States, unlike our study, revealed taxonomical differences between diabetics and non-diabetics, however, the differences in age and BMI between both groups could influence their results. The lack of high differences in our study diverges from what was found in studies from uncontrolled diabetics (Farina et al., 2019). This disagreement, together with the results of Long et al. (2017), which found differences in microbiome composition when comparing diabetics with adequate and poor metabolic control, highlights the impact of diabetes being controlled in the oral microbiome. Therefore, our results indicate that the relationship between the oral microbiome and diabetes, involving oral diseases, would be mainly related to the lifestyle or the consequence of lack of control of the disease rather than to having diabetes. Thus, the major suggestion from our study is that future studies relating to T2D with oral microbiome should include dietary and lifestyle habits as two main sets of variables to contrast. In addition, more studies on the oral microbiome of controlled diabetics are necessary for optimal comparable datasets.

## Data Availability Statement

The datasets presented in this study can be found in online repositories. The names of the repository and accession number can be found below: https://www.ncbi.nlm.nih.gov/, PRJNA679485.

## Ethics Statement

The studies involving human participants were reviewed and approved by the Centro Hospitalar de Vila Nova de Gaia/Espinho’s Ethics Committee. The patients/participants provided their written informed consent to participate in this study.

## Author Contributions

AA-S, AB-P, MG-V, LP-P, and DM-M conceived and designed the study. AA-S and DM-M collected the samples. AA-S and MG-V performed laboratory assays. AA-S and LP-P performed bioinfomatic analysis. AA-S performed statistical analysis and wrote the draft of the manuscript. AA-S, MG-V, and LP-P interpreted the results. MG-V, LP-P, and AB-P supervised the work and revised and contributed to the final manuscript. AB-P contributed with resources and funding. All the authors read and approved the final manuscript.

## Conflict of Interest

The authors declare that the research was conducted in the absence of any commercial or financial relationships that could be construed as a potential conflict of interest.

## Abbreviations

ASV: amplicon sequence variant
bp: Base pairs
T2DM: Type 2 Diabetes Mellitus.
