# Supplementary material for: Characterization of the Oral Microbiome of Medicated Type-2 Diabetes Patients
Source: Front Microbiol. 2021 Feb 5;12:610370. doi: 10.3389/fmicb.2021.610370 (PMC7892904; doi:10.3389/fmicb.2021.610370)
Supplement: Supplementary file 1 [file Data_Sheet_2.docx]

**Characterization of the oral microbiome of medicated type 2 diabetes patients**

Ana Almeida-Santos^1,2^, Daniela Martins-Mendes^3,4,5,6^, Magdalena Gayà-Vidal^1#^, Lucía Pérez-Pardal^1#^, Albano Beja-Pereira^1,2,7,#*^

**Supplementary Material**

**Supplementary Table S3.** Diabetes patients’ medication divided into categories

|  | **Medication** | | | | | | | | | | |
| --- | --- | --- | --- | --- | --- | --- | --- | --- | --- | --- | --- |
| **Sample** | Metformin | PPIs | DPP4 inhibitor | SGLT2 inhibitor | Levothyroxine | Sulfonylurea | Statin | Antidepressants | Statin + Metformin | DPP4 + Metformin | Insulin |
| **DM3** | Yes | Yes | No | No | No | No | No | Yes | No | No | Yes |
| **DM4** | No | No | No | No | No | No | No | No | No | No | Yes |
| **DM6** | Yes | No | No | No | Yes | No | No | No | No | No | No |
| **DM7** | Yes | Yes | No | No | No | No | No | No | No | No | No |
| **DM8** | Yes | No | No | No | No | No | No | Yes | No | No | Yes |
| **DM11** | No | Yes | Yes | No | Yes | No | Yes | Yes | No | No | No |
| **DM12** | No | No | Yes | No | No | No | Yes | No | No | No | No |
| **DM13** | Yes | No | Yes | Yes | No | No | Yes | No | Yes | Yes | No |
| **DM14** | Yes | No | No | Yes | No | No | Yes | No | Yes | No | No |
| **DM15** | Yes | No | Yes | Yes | No | No | No | No | No | Yes | No |
| **DM16** | Yes | No | No | Yes | Yes | No | Yes | Yes | Yes | No | No |
| **DM18** | Yes | No | Yes | No | No | Yes | No | No | No | Yes | No |
| **DM19** | Yes | No | No | Yes | No | No | Yes | No | Yes | No | Yes |
| **DM20** | Yes | No | No | No | No | No | No | No | No | No | No |
| **DM21** | Yes | No | Yes | No | No | Yes | Yes | No | Yes | Yes | No |
| **DM22** | Yes | Yes | Yes | No | No | No | Yes | No | Yes | Yes | Yes |
| **DM23** | Yes | No | Yes | No | No | Yes | No | No | No | Yes | No |
| **DM24** | No | No | No | No | No | No | Yes | No | No | No | Yes |
| **DM26** | Yes | No | Yes | No | No | No | No | Yes | No | Yes | No |
| **DM28** | Yes | No | Yes | Yes | No | No | No | No | No | Yes | No |
| **DM29** | No | No | No | No | No | No | No | Yes | No | No | Yes |
| **DM31** | Yes | No | Yes | No | No | No | No | No | No | Yes | Yes |

Yes – Intake of this type of medication
 No – No intake of this type of medication

**Supplementary table S4**. Nutrients consumption and energy intake of both control and diabetes individuals.

|  | Sample | Calories (Kcal) | Protein (g) | Carbohydrates (g) | Total Fat (g) | Saturated fat (g) | Monounsaturated Fat (g) | Polyunsaturated Fat (g) | Sugar (g) |
| --- | --- | --- | --- | --- | --- | --- | --- | --- | --- |
| Controls | C1 | 1632.6 | 82.92 | 201.1 | 55.56 | 16.2 | 26.17 | 08.07 | 99.1 |
|  | C2 | 2443.47 | 89.41 | 244.86 | 104.81 | 34.76 | 51.01 | 11.9 | 87.1 |
|  | C3 | 3570.95 | 196.78 | 361.64 | 154.13 | 43.53 | 73.09 | 25.88 | 123.03 |
|  | C5 | 1996.15 | 112.18 | 217.51 | 74.66 | 18.47 | 35.07 | 13.94 | 75.89 |
|  | C7 | 2057.65 | 75.95 | 246.93 | 80.41 | 22.53 | 38.1 | 13.39 | 106.35 |
|  | C8 | 2356.24 | 102.28 | 277.51 | 87.84 | 20.46 | 42.76 | 17.26 | 117.1 |
|  | C9 | 818.85 | 50.34 | 92.6 | 27.77 | 7.92 | 11.24 | 5.81 | 34.22 |
|  | C12 | 2346.68 | 113.38 | 263.82 | 86.51 | 29.28 | 34.71 | 14.95 | 82.29 |
|  | C15 | 1238.57 | 76.96 | 96.47 | 55.31 | 15.34 | 25.32 | 9.94 | 30.12 |
|  | C16 | 2334.93 | 108 | 287.74 | 89.63 | 23.36 | 39.74 | 18.86 | 133.9 |
|  | C17 | 1824.4 | 75.43 | 206.24 | 71.89 | 21.39 | 31.29 | 13.53 | 109.78 |
|  | C19 | 1078.7 | 75.31 | 92.82 | 46.11 | 10.94 | 23.26 | 7.77 | 49.49 |
|  | C20 | 1483.63 | 73.78 | 150.9 | 67.33 | 15.18 | 38.52 | 8.6 | 70.62 |
|  | C21 | 1777.32 | 77.45 | 222.43 | 67.28 | 19.05 | 32.76 | 10.23 | 71.13 |
|  | C23 | 2225.15 | 111.42 | 229.22 | 103.07 | 24.19 | 51.45 | 19.47 | 127.72 |
|  | C24 | 2203.08 | 105 | 249.67 | 89.67 | 26.39 | 41.81 | 13.77 | 97.81 |
|  | C26 | 2003.19 | 89.67 | 215.77 | 66.17 | 16.58 | 32.13 | 11.47 | 93.33 |
|  | C27 | 2064.29 | 98.35 | 268.75 | 61.94 | 13.39 | 31.19 | 12.1 | 121.11 |
|  | C28 | 1882.09 | 89.44 | 238.91 | 57.45 | 13.34 | 26.92 | 11.83 | 115.78 |
|  | C31 | 1905.18 | 91.36 | 261.22 | 59.99 | 17.46 | 24.98 | 11.19 | 113.79 |
|  | C32 | 2195.69 | 77.43 | 296.86 | 75.92 | 15.87 | 41.64 | 12.33 | 97.76 |
|  | C50 | 2698.95 | 160.95 | 264.56 | 108.61 | 36.49 | 46.34 | 16.14 | 141.04 |
|  | C51 | 1781.66 | 111.26 | 163.29 | 72.97 | 24.72 | 30.58 | 10.9 | 58.98 |
|  | C98 | 1842.16 | 84.36 | 232.01 | 42.28 | 14.01 | 16.32 | 6.94 | 113.19 |
| Diabetes patients | DM3 | 2690.2 | 152.6 | 317.77 | 97.74 | 21.51 | 50.96 | 16.65 | 134.75 |
|  | DM6 | 3286.08 | 166.16 | 281.34 | 151.68 | 30.8 | 82.72 | 25.19 | 101.79 |
|  | DM7 | 1922.56 | 111.7 | 256.45 | 56.23 | 15.36 | 23.78 | 10.64 | 83.61 |
|  | DM8 | 1689.56 | 64.75 | 226.94 | 61.1 | 16.81 | 29.13 | 10.09 | 61.04 |
|  | DM11 | 1850.6 | 105.74 | 203.71 | 72.06 | 22.18 | 32.49 | 10.67 | 68.61 |
|  | DM12 | 4037.56 | 211.27 | 406.89 | 156.55 | 40.72 | 68.76 | 33.25 | 150.98 |
|  | DM13 | 1992.2 | 90.09 | 193.79 | 74.58 | 21.1 | 35.92 | 10.84 | 85.6 |
|  | DM14 | 1641.42 | 80.57 | 149.24 | 60.57 | 15.68 | 29.53 | 9.86 | 58.31 |
|  | DM15 | 2119.9 | 91.21 | 298.54 | 67.84 | 17.59 | 28.1 | 16.22 | 117.06 |
|  | DM16 | 1333.11 | 61.69 | 168.61 | 49.39 | 13 | 24.19 | 7.44 | 104.11 |
|  | DM18 | 1777.16 | 95.79 | 166.43 | 82.7 | 21.82 | 35.22 | 17.4 | 64.74 |
|  | DM19 | 2003.51 | 95.25 | 207.99 | 81.85 | 17.16 | 43.78 | 14.14 | 45.45 |
|  | DM20 | 2313.78 | 126.64 | 224.67 | 77.69 | 22.35 | 34.4 | 13.38 | 74.9 |
|  | DM21 | 1390.72 | 59.87 | 138.82 | 43.84 | 15.14 | 17.79 | 07.04 | 33.68 |
|  | DM22 | 1873.87 | 111.17 | 220.16 | 63.78 | 19.19 | 27.68 | 10.47 | 94.99 |
|  | DM23 | 2712.02 | 120.54 | 282.13 | 101.46 | 28.11 | 46.28 | 18.55 | 91.7 |
|  | DM24 | 1713.71 | 116.22 | 163.68 | 64.45 | 19.57 | 28.45 | 9.47 | 72.71 |
|  | DM26 | 1453.09 | 78.65 | 184.29 | 47.09 | 13.44 | 20.54 | 8.27 | 64.93 |
|  | DM27 | 1765.82 | 95.89 | 207.96 | 62.73 | 17.09 | 29.95 | 10.02 | 66.45 |
|  | DM28 | 2203.95 | 131.69 | 208.05 | 94.17 | 25.99 | 41.92 | 17.32 | 39.48 |
|  | DM29 | 2861.43 | 140.15 | 269.23 | 139.87 | 29.22 | 67.65 | 32.23 | 94.13 |
|  | DM31 | 1960.28 | 116.58 | 232.19 | 66.68 | 18.21 | 30.03 | 11.8 | 78.93 |
|  | DM35 | 3888.37 | 150.37 | 439.53 | 170.42 | 46.08 | 83.39 | 28.15 | 168.17 |
|  | DM36 | 1663.02 | 76.86 | 164.99 | 62.67 | 16.72 | 29.7 | 11.25 | 60.27 |
|  | DM4 | 2851.77 | 125.76 | 291.07 | 118.29 | 21.8 | 66.64 | 21.08 | 84.34 |

**Supplementary Table S6.** Taxa that were significantly different between the control and diabetes group at class, genus and species level.

|  | **Frequency (%)** | | **Range** | | **Frequency (%)** | **Range** | **P-value (Mann-Whitney test)** | |
| --- | --- | --- | --- | --- | --- | --- | --- | --- |
| **Class level** | **Control** |  | | **Diabetes** | |  | |  |
| *Synergistia* | 0.248 | 0-0.010 | | 0.006 | | 0-0.0009 | | <0.001* |
| *Deltaproteobacteria* | 0.021 | 0-0.002 | | 0.000 | | 0-0 | | 0.013 |
| *Betaproteobacteria* | 4.371 | 0-0.278 | | 9.189 | | 0-0.323 | | 0.033 |
| *Spirochaetes* | 1.071 | 0-0624 | | 0.348 | | 0-0.316 | | 0.035 |
| *Mollicutes* | 0.184 | 0-0.143 | | 0.074 | | 0-004 | | 0.043 |
| **Genus level** |  |  | |  | |  | |  |
| ***TG5*** | 0.246 | 0-0.010 | | 0.006 | | 0-0.001 | | <0.001* |
| *Mycoplasma* | 0.142 | 0-0.008 | | 0.008 | | 0-0.002 | | <0.001 |
| *Granulicatella* | 0.647 | 0-0.016 | | 1.020 | | 0-0.018 | | 0.002 |
| *Acidaminobacteraceae* | 0.041 | 0-0.004 | | 0.000 | | 0-0 | | 0.003 |
| *Filifactor* | 0.235 | 0-0.010 | | 0.043 | | 0-0.004 | | 0.006 |
| *Mogibacteriaceae* | 0.808 | 0-0.400 | | 0.326 | | 0-0.010 | | 0.007 |
| *Enterobacteriaceae* | 4.198 | 0-0.0003 | | 0.020 | | 0-0 | | 0.017 |
| *Gluconacetobacter* | 0.153 | 0-0.008 | | 0.039 | | 0-009 | | 0.022 |
| *Neisseria* | 3.045 | 0-0.256 | | 7.313 | | 0-0.291 | | 0.039 |
| *Tissierellaceae* | 0.022 | 0-0.002 | | 0.000 | | 0-0 | | 0.025 |
| *Peptococcus* | 0.180 | 0-0.010 | | 0.049 | | 0-0.004 | | 0.020 |
| *Treponema* | 1.070 | 0-0.062 | | 0.348 | | 0-0.031 | | 0.035 |
| *Bacteroidales* | 0.196 | 0-0.009 | | 0.039 | | 0-0-003 | | 0.030 |
| *Oribacterium* | 1.125 | 0-0.030 | | 0.693 | | 0-0.036 | | 0.035 |
| **Species level** |  |  | |  | |  | |  |
| ***TG5 spp****.* | 0.246 | 0-0.010 | | 0.006 | | 0-0.001 | | <0.001* |
| *Mycoplasma spp.* | 0.142 | 0-0.079 | | 0.008 | | 0-0.002 | | <0.001 |
| *Granulicatella spp* | 0.647 | 0-0.016 | | 1.020 | | 0-0.175 | | 0.002 |
| *Streptococcus anginosus* | 0.132 | 0-0.009 | | 0.007 | | 0-0.002 | | 0.002 |
| *Acidaminobacteraceae* | 0.041 | 0-0.004 | | 0.000 | | 0-0 | | 0.003 |
| *Bulleidia spp.* | 0.073 | 0-0.011 | | 0.000 | | 0-0 | | 0.007 |
| *Filifactor spp.* | 0.235 | 0-0.010 | | 0.043 | | 0-0.004 | | 0.006 |
| *Mogibacteriaceae* | 0.808 | 0-0.400 | | 0.326 | | 0-0.010 | | 0.007 |
| *Enterobacteriaceae* | 4.198 | 0-0.0003 | | 0.020 | | 0-0 | | 0.017 |
| *Gluconacetobacter spp.* | 0.153 | 0-0.080 | | 0.039 | | 0-0.009 | | 0.022 |
| *Porphyromonas spp.* | 2.677 | 0-0.086 | | 4.490 | | 0.0026-0.170 | | 0.025 |
| *Neisseria* | 3.045 | 0-0.256 | | 7.313 | | 0-0.291 | | 0.023 |
| *Treponema socranskii* | 0.130 | 0-0.09 | | 0.040 | | 0-0.005 | | 0.019 |
| *Rothia dentocariosa* | 1.047 | 0-0.055 | | 0.375 | | 0-0.010 | | 0.016 |
| *Peptococcus spp.* | 0.180 | 0-0.010 | | 0.049 | | 0-0.004 | | 0.020 |
| *Tissierellaceae* | 0.022 | 0-0.002 | | 0.000 | | 0-0 | | 0.025 |
| *Treponema spp.* | 0.774 | 0-0.050 | | 0.248 | | 0-0.028 | | 0.025 |
| *Bacteroidales* | 0.196 | 0-0.009 | | 0.039 | | 0-0-003 | | 0.030 |
| *Neisseria bacilliformis* | 0.036 | 0-0.005 | | 0.000 | | 0-0 | | 0.048 |
| *Oribacterium spp.* | 1.125 | 0-0.030 | | 0.693 | | 0-0.361 | | 0.035 |

*p-values that remained significant after Bonferroni correction for multitesting.

**In bold**: significant p_value was also significant with the ANCOM analysis


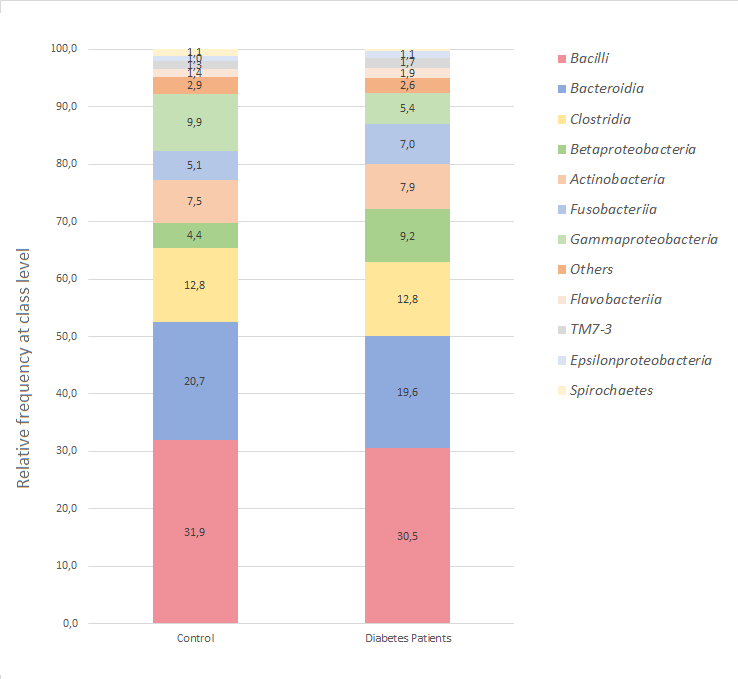


**Supplementary Figure S1.** Relative frequency of the ten most-abundant taxa found at the class level in both control and diabetes groups.


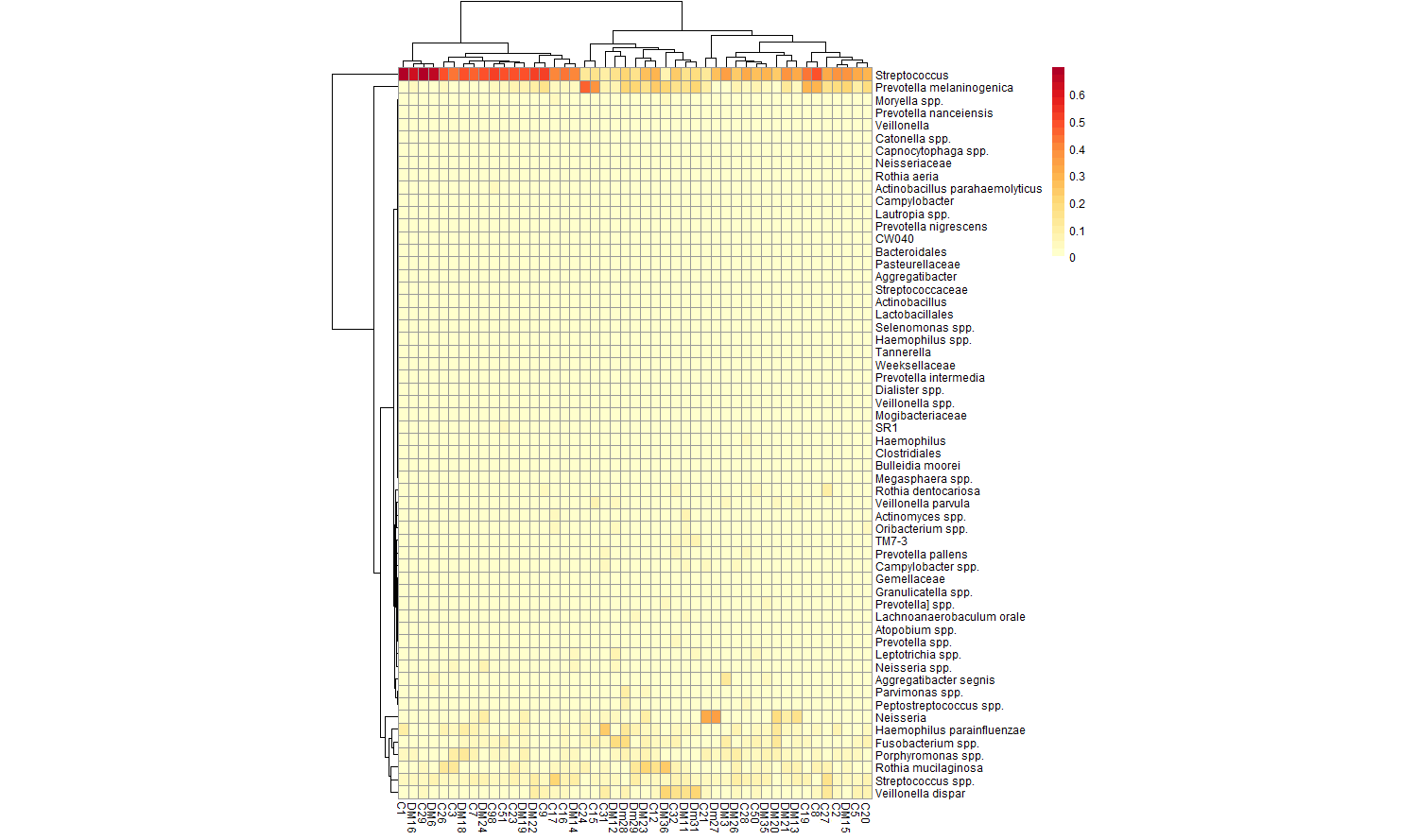


**Supplementary Figure S2.** Heatmap of the relative abundance of the taxa that were present in more than 15% of the samples, and the corresponding hierarchical clustering. Each column of the heatmap represents the individuals from control (C) and diabetes (DM) groups and the rows are the taxa up to the species level. The vertical color bar on the right side of the graph defines the relative abundance of each taxon.

B)

A)


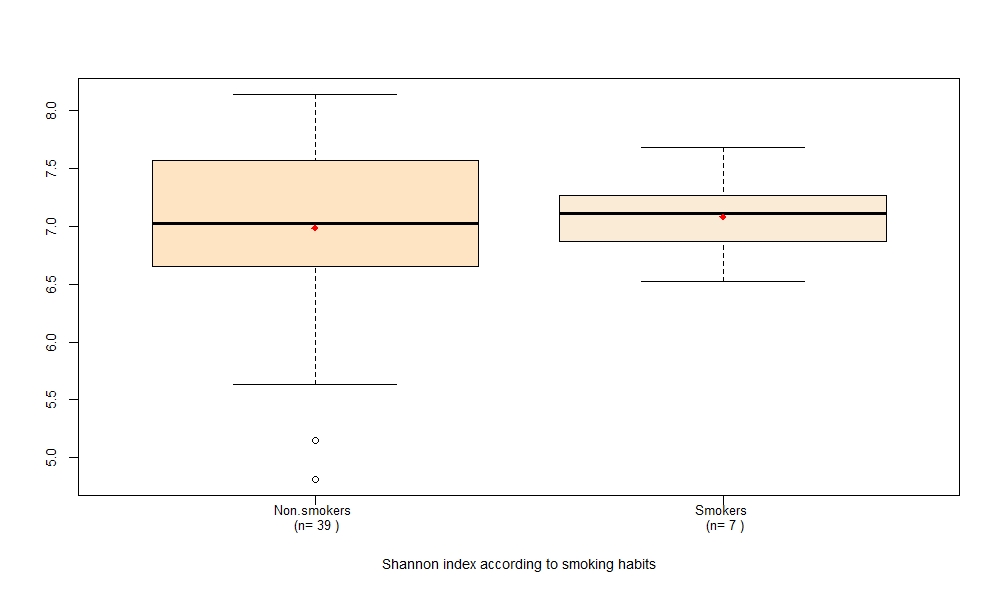

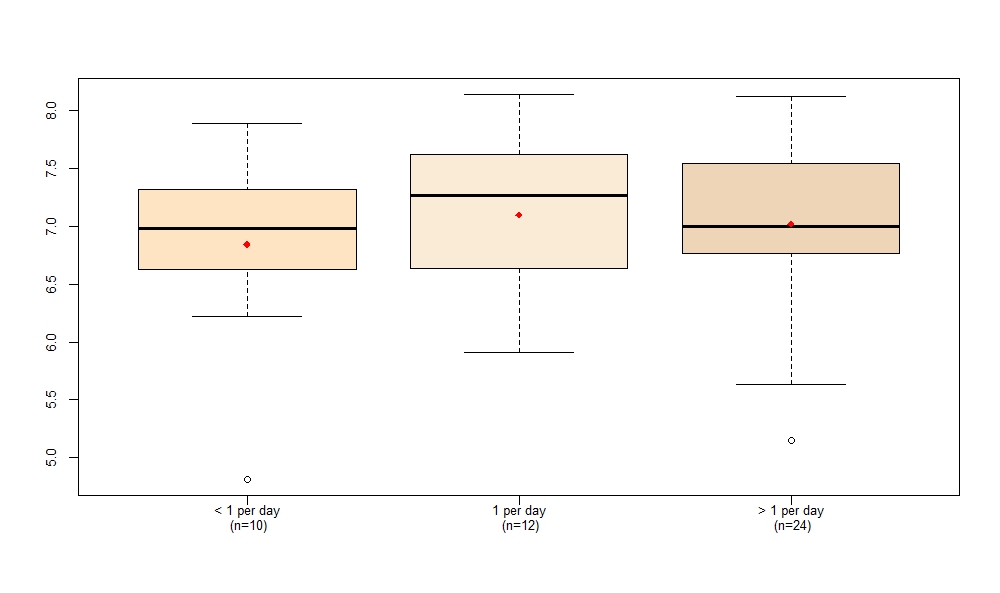


**Supplementary Figure S3.** Boxplot charts depicting the distribution of the Shannon index for A) teeth brushing habits, and B) smoking habits. Red dot stands for the mean of each group


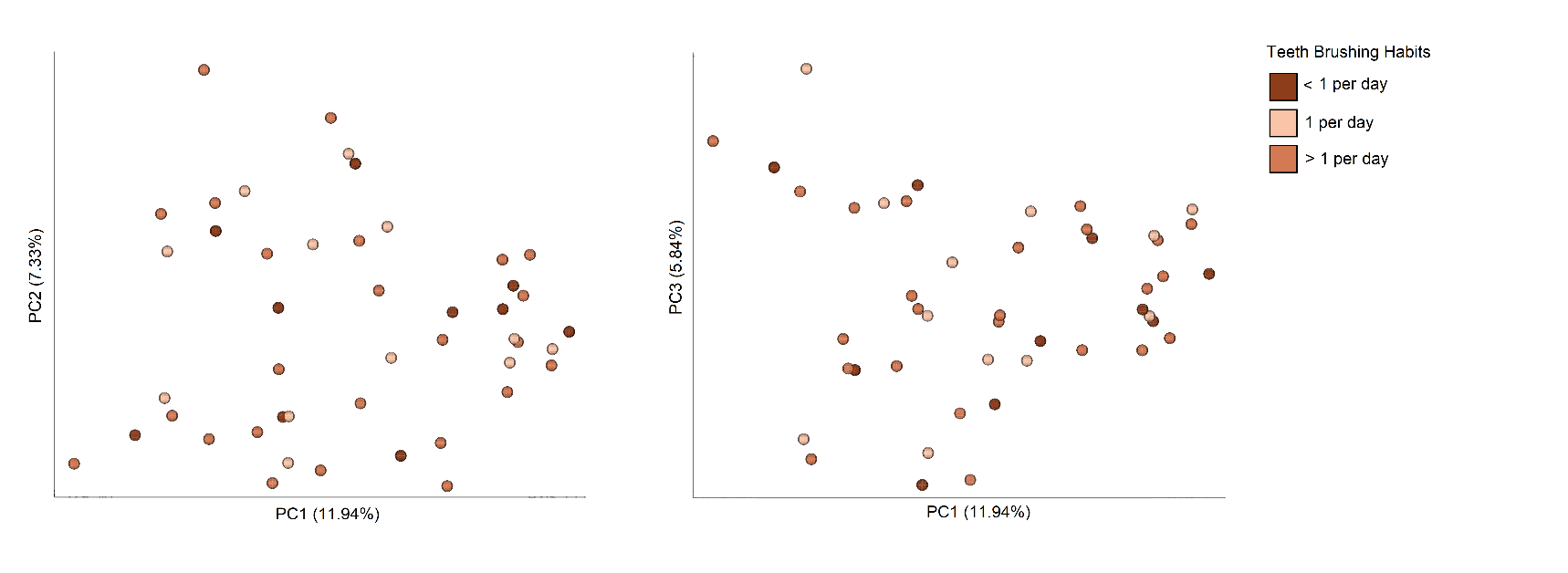


A)

B)

**Supplementary** **Figure S4.** PCoA plots showing the A) first and second, and B) first and third principal components and the percentage of the total variance that they explain based on the Bray Curtis dissimilarity matrix. Each point represents one individual, with color and symbol indicating the brushing teeth habits.


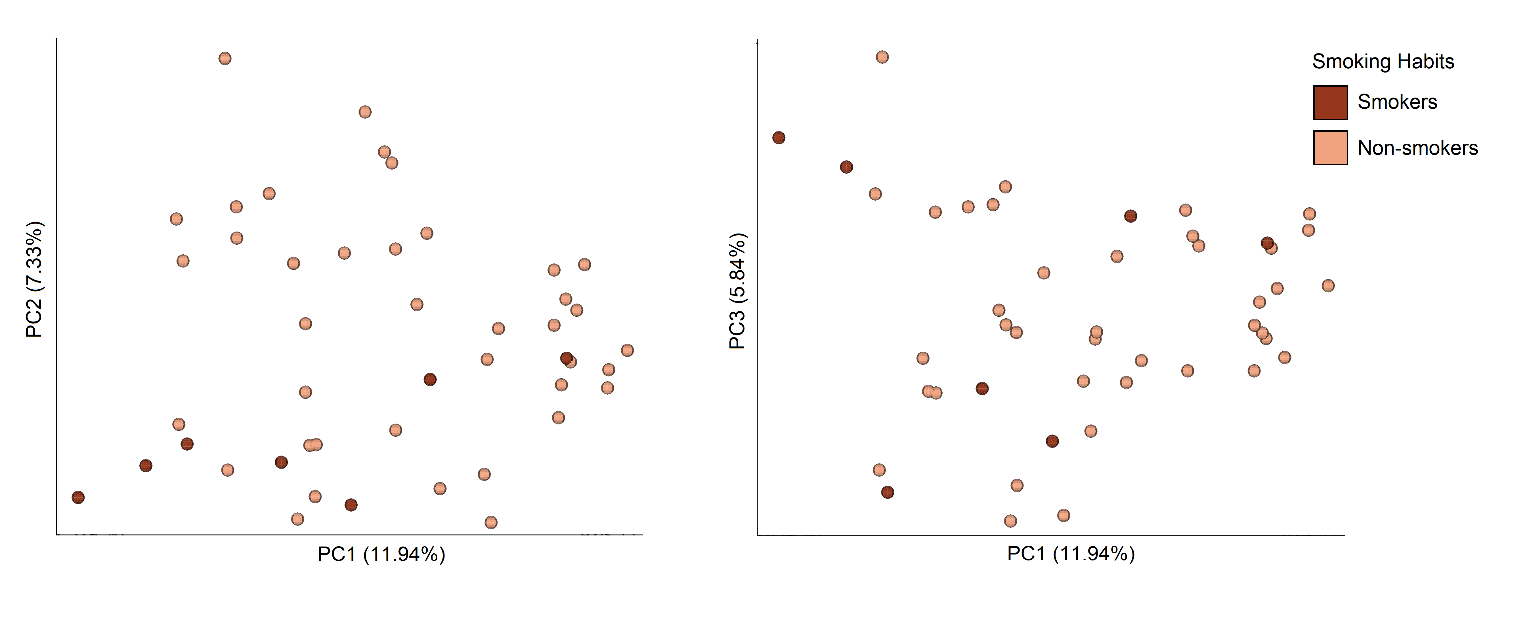


A)

B)

**Supplementary Figure S5.** PCoA plots showing the A) first and second, and B) first and third principal components and the percentage of the total variance that they explain based on the Bray Curtis dissimilarity matrix. Each point represents one individual, with color and symbol indicating the smoking habits.


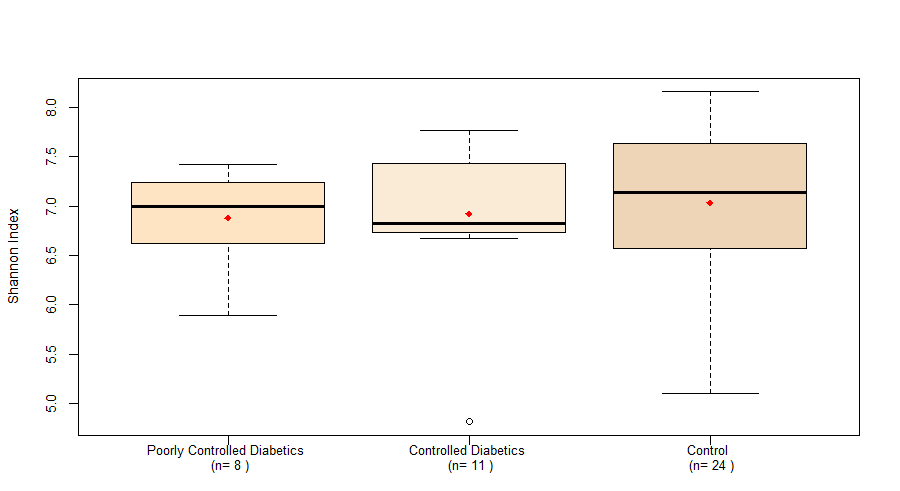
**Supplementary Figure S6.** Boxplot charts depicting the distribution of the Shannon index regarding HbA1c categories (Poorly controlled diabetics: HbA1c >7; Controlled Diabetics: HbA1c < 7) and the control group. Red dot stands for the mean of each group.


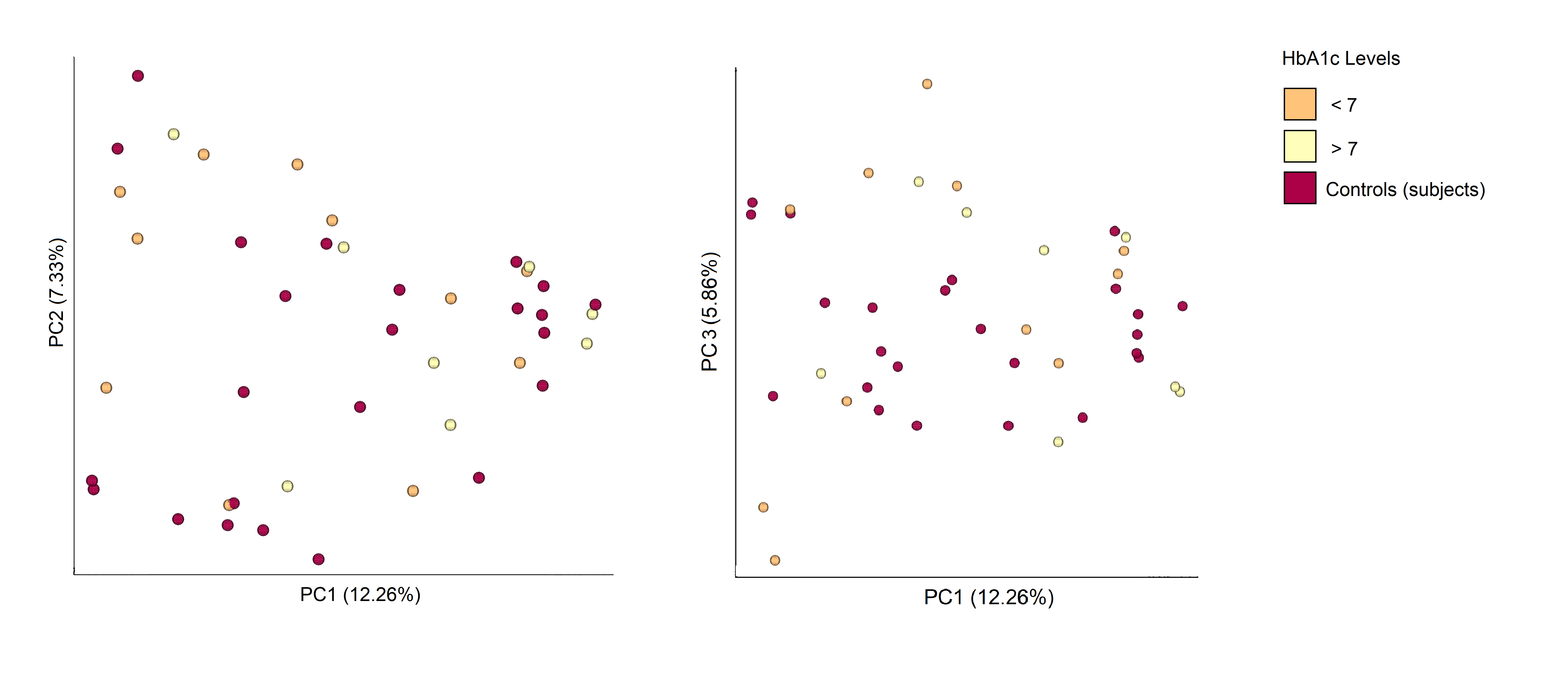


**Supplementary Figure S7.** PCoA plots showing the A) first and second, and B) first and third principal components and the percentage of the total variance that they explain based on the Bray Curtis dissimilarity matrix. Each point represents one individual, with color and symbol indicating the HbA1c categories and the control subjects.
